# Interaction Between Composite Dietary Antioxidant Index and Alcohol Consumption on Cardiovascular Diseases: NHANES 2005–2018

**DOI:** 10.1002/clc.70274

**Published:** 2026-03-07

**Authors:** Yingjie Zhu, Lili Zheng, Jia Bing, Xiaoyu Teng, Pengkai Hao, Ping Song, Lixin Wan

**Affiliations:** ^1^ Jilin Provincial Institute of Population Life Science and Technology (Jilin Provincial Reproductive Health Hospital) Changchun P. R. China; ^2^ Jilin Maternal and Child Health Hospital (Jilin Obstetrics Quality Control Center) Changchun P. R. China

**Keywords:** alcohol consumption, cardiovascular diseases (CVDs), composite dietary antioxidant index (CDAI), NHANES, oxidative stress

## Abstract

**Background:**

Cardiovascular diseases (CVDs) are a group of heart and blood vessel disorders and the leading causes of death worldwide. Few studies have focused on whether there is an interaction between the Composite Dietary Antioxidant Index (CDAI) combined with alcohol consumption on CVDs. We aimed to explore the association between CDAI, alcohol consumption, and CVDs, and whether there was an interaction.

**Methods:**

A total of 29459 participants aged over 20 years from the National Health and Nutrition Examination Survey (NHANES) in 2005–2018 were involved in the study. Six dietary factors were selected to score the CDAI. The association between CDAI, alcohol consumption, and CVDs were analyzed using binary logistic regression. Subgroup analysis and interaction tests were used to investigate whether this association was stable across populations.

**Results:**

The interaction between CDAI and alcohol consumption in relation to CVDs was observed. There was a statistically significant increased prevalence of CVDs in the CDAI 2 combined never‐drinking subgroups and in the CDAI 1 combined never‐drinking subgroups compared with the CDAI 3 combined moderate drinking group. Low CDAI levels were significantly and positively linked to CVDs prevalence within the never‐drinking subgroup.

**Conclusion:**

The interaction between CDAI and alcohol consumption was found in our study. High levels of CDAI combined with moderate alcohol consumption may reduce the odds of CVDs.

## Summary

- This study holds significance as the first to investigate the interaction between CDAI and alcohol consumption on CVDs.
- Higher CDAI was associated with a lower prevalence of CVDs.
- It is advisable for individuals to enhance their personal CDAI levels by incorporating more antioxidant nutrients into their diet, thereby mitigating the detrimental effects of CVDs on the body.

## Graphical abstract

Figure 1 Flowchart for the study design and participants. An initial sample of 39 636 participants was assessed. Participants were sequentially excluded: (1) missing data on CDAI components; (2) missing data on covariates; and (3) currently pregnant. A final sample of 29 459 eligible individuals was included in our analysis.

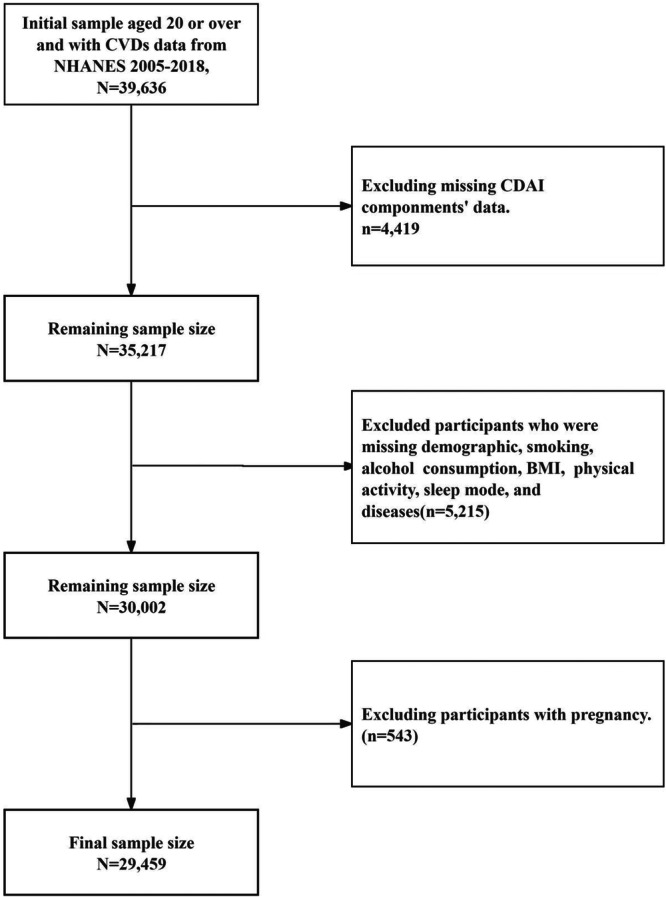

## 1 Introduction

Cardiovascular diseases (CVDs) are a group of heart and blood vessel disorders that include coronary heart disease, peripheral artery disease, rheumatic heart disease, and so on [1]. The structure and function of the body's blood vessels undergo a series of changes due to aging, including atherosclerosis of the main arteries, increased pulse pressure and the formation of blood clots, and so on, which in turn leads to an increased risk of developing CVDs [2]. They have collectively remained the leading cause of death worldwide, and substantially contributed to loss of health and excess health system costs [3]. With the dramatic acceleration of global population aging and the enormous burden that CVDs place on patients, including but not limited to elderly patients, caregivers, and healthcare systems, CVDs are undoubtedly one of the most serious public health problems we will face in the 21st century.

The potential mechanisms underlying the development of CVDs have been shown in previous studies to be closely related to oxidative stress (OS) and the ensuing inflammatory response [4]. OS is a pathological condition caused by an imbalance between the organism's antioxidant system and reactive oxygen species (ROS) such as free radicals [5, 6]. Within the cells of the body, the balance is tightly controlled, and when it is disrupted, it may cause a variety of undesirable diseases, including CVDs [7, 8, 9]. Dietary antioxidants are known to have an interventional role in adverse health effects such as oxidative stress and chronic inflammation [10, 11]. A variety of nutritional nutrients are considered to be excellent antioxidant food sources for the organism and influence the level of oxidative stress in the organism [12, 13, 14]. Since multiple antioxidant nutrients are commonly combined in the same food and there are complex biological interactions between them, the role of a single nutrient in influencing CVDs may not be accurate. The Composite Dietary Antioxidant Index (CDAI), developed by Wright et al. in 2004, represents a composite score of multiple dietary antioxidants (including vitamins A, C, E, selenium, zinc, and carotenoids) that represents an individual's overall nutritional antioxidant intake [15]. Previous studies have shown that the CDAI was associated with a variety of adverse health outcomes, including depression [16], all‐cause mortality [17], hypertension [18], and colorectal cancer [19]. One of the most serious risk factors for disease and death globally is alcohol. It has been demonstrated that consuming alcohol has complicated, and occasionally contradictory, links to CVDs [20]. The relationship between alcohol consumption and CVDs has been shown to be complex in hundreds of previous studies [21, 22, 23]. Since alcohol consumption has potentially beneficial effects on some CVDs outcomes, the association between alcohol consumption and CVDs is controversial. In addition, the interaction of CDAI with alcohol consumption on CVDs is unknown. Therefore, we conducted a cross‐sectional study using data from the National Health and Nutrition Examination Survey (NHANES) to assess the association between CDAI, alcohol consumption, and CVDs.

## 2 Materials and Methods

### 2.1 Data Source and Study Participants

**FIGURE 1:**
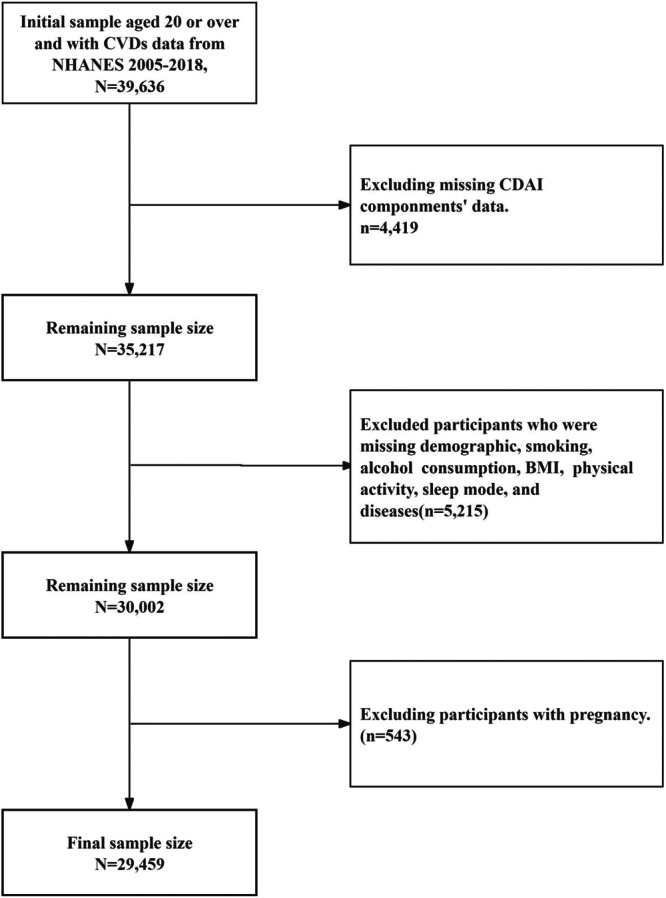
Flowchart for the study design and participants.

The information for this study was gathered from the 2005 to 2018 cycles of the NHANES. The Centers for Disease Control and Prevention (CDC) performed the NHANES, which aims to evaluate the health and nutritional status of the United States (U.S.) population. The survey used a complex probability sampling strategy, collecting information through standardized interviews, physical examinations, and biological sample testing. The National Centre for Health Statistics study ethical review board authorized the NHANES methods, and all participants gave informed consent before participating in the surveys. For additional information on the NHANES’ techniques and procedures, please visit the official website at http://www.cdc.gov/nchs/nhanes.htm. An initial sample of 39 636 participants aged 20 years or older with available CVDs data from NHANES 2005–2018 was assessed for eligibility. Participants were sequentially excluded based on the following strict criteria: (1) missing data on CDAI components (*n* = 4419); (2) missing data on covariates, including demographics, smoking status, alcohol consumption, BMI, physical activity, sleep mode, and history of diseases (*n* = 5215); and (3) currently pregnant (*n* = 543). After these exclusions, a final sample of 29 459 eligible individuals was included in our analysis (Figure 1).

### 2.2 Dietary Information

The survey's Nutrition Methodology Working Group conducted 24‐h dietary recall interviews at the mobile examination center (MCE) to obtain all dietary information. The participants responded to two recall surveys, the initial recall was conducted in the MEC, while the second took place 3–10 days later via telephone. We used data from two surveys to calculate the CDAI as utilizing dietary intake data from two non‐consecutive days was considered to be more accurate than relying on a single day's data [24, 25]. The calculation of CDAI was based on the concentration of antioxidant components in foods and the antioxidant capacity weight of each component. It was composed of six dietary antioxidants: zinc, selenium, carotenoids, vitamin A, C, and E using Wright's proposed measuring technique [15]. We subtracted the universal mean and divided the results by its worldwide standard deviation from the six dietary minerals and vitamins as follows:

$$\text{CDAI}\;=\sum_{i=1}^{6}\frac{x_{i}-u_{i}}{s_{i}}$$

To group the CDAIs according to their thirds, we define the first tertile of the CDAI as CDAI 1, and correspondingly define the second and third tertiles of the CDAI as CDAI 2, CDAI 3.

### 2.3 Cardiovascular Diseases (CVDs)

Similar to previously published articles based on NHANES [26, 27], the diagnosis of cardiovascular disease was mainly determined by an individual interview questionnaire for standardized medical conditions. We used the medical condition questionnaire (MCQ) “MCQ160B,” “MCQ160C,” “MCQ160D,” “MCQ160E,” and “MCQ160F” variables were used to diagnose congestive heart failure (CHF), coronary heart disease (CHD), angina, heart attack, and stroke, respectively. Participants were asked, “Has a doctor or other health professional ever told you that you have CHF/CHD/angina/heart attack/stroke?” If a participant answered “yes” to any of these questions, they were considered to have CVDs. The “didn't know” participants would be excluded.

### 2.4 Covariates

Covariates included age (20–39, 40–59, or ≥ 60 years), race (Non‐Hispanic White, or others), sex (male, or female), education level (below high school, high school, or above high school), marital status (married/living with partner, or widowed/divorced/separated/never married), physical activity (active, or inactive), body mass index (BMI) (normal, overweight, or obese), smoking status (current smoker, former smoker, or never), alcohol consumption (heavy drinker, moderate drinker, or never), sleep mode (poor, moderate, or healthy), hypertension (yes, or no), and diabetes (yes, or no).

Physical activity is divided into two groups according to World Health Organization (WHO) guidelines [28]: individuals who perform more than 149 min of moderate physical activity, more than 74 min of vigorous physical activity, or more than 599 metabolic equivalent (MET) minutes per week are assigned to the active group; the rest of the population is assigned to the inactive group. BMI ≥ 30.0 kg/m^2^ was defined as obese, 25.0–29.9 kg/m^2^ was defined as overweight, and BMI < 25.0 kg/m^2^ as normal. Smoking status was divided into three groups for analysis. Participants were classified as current smokers if they had smoked at least 100 cigarettes and continued to smoke on a daily or intermittent basis. Former smokers were classified as those who had previously smoked at least 100 cigarettes but were no longer smoking. Non‐smokers were individuals who have never smoked more than 100 cigarettes throughout their lifetime [29]. The alcohol consumption was categorized into three groups, with 0.0 g/day defined as a non‐drinker, 0.1–30.0 g/day defined as a moderate drinker, and > 30.0 g/day defined as a heavy drinker [30]. Sleep mode is assessed using three questions [31, 32]. (1) Sleep duration: “How many hours do you usually sleep on weekdays or weekday nights.” Recorded sleep duration was categorized as short (less than 7 h per night), normal (7–9 h per night) and long (more than 9 h per night), with normal sleep duration scored as 0 point and short/long sleep duration scored as 1 point. (2) Trouble sleeping: “Have you ever told your doctor or other health professional that you have a trouble sleeping,” with a “yes” answer scored as 1 point and a “no” answer scored as 0 points. (3) Sleep disorder: “Have you ever been told by a doctor or other health care professional that you have a sleep disorder” or “In the past month, how often did you feel excessively or overly sleepy during the day.” The former was scored as 1 for “yes” and 0 for “no.” The latter was divided into two categories: normal (less than or equal to four times per month) and sleepy (more than four times per month), with normal being scored as 0 and sleepy as 1. The scores for the above three questions were added together to give a total score between 0 and 3. A score of 0 was regarded as a healthy sleep mode, a score of 1 was regarded as a moderate sleep mode, and a score of ≥ 2 was regarded as a poor sleep mode. Besides, participants were considered to have a history of hypertension, and diabetes if they self‐reported a physician diagnosis of such diseases.

### 2.5 Statistical Analyses

Weighted means and standard error (SE) represented continuous variables, while categorical variables were conveyed as counts and percentages. To discern variations in baseline traits between participants with and without CVDs, One‐Way ANOVA and chi‐square test were utilized for continuous and categorical variables, respectively. We used three weighted logistic regression models to evaluate the relationship between the CDAI and CVDs. Model 1 was adjusted for nothing, Model 2 adjusted for age, sex, and race, Model 3 additionally adjusted for education level, marital status, smoking status, alcohol level, physical activity, BMI, sleep mode, diabetes, and hypertension based on Model 2. *P*‐interaction between CDAI and each stratified variable was tested and subgroup analysis was used to further validate the stability of results. All statistical analyses of the study population were done under complex weighting given the complexity of the sampling method. All statistical analyses were performed by IBM SPSS 24.0 software. Statistical significance was defined as a two‐tailed *P* value of < 0.05, and the interaction was considered suggestive with *p* < 0.10 [33, 34].

## 3 Result

The final analysis included 29 459 subjects, 3082 with CVDs and 26 377 without. Table 1 depicts the basic features of the study population according to CDAI tertiles. There were significant differences (*p* < 0.05) in sex, age, race, smoking status, alcohol level, education level, physical activity, marital status, BMI, sleep mode, diabetes, hypertension, and CVDs between the three tertile groups. In contrast, the CDAI 3 group tended to be characterized by a younger age, male gender, active physical activity, a higher chance of being Non‐Hispanic White, married/living with a partner, having a higher education level, and consuming more alcohol, having a lower prevalence of diabetes, hypertension and CVDs. Additionally, this group was less likely to smoke than individuals in lower CDAI tertiles.

**TABLE 1:** Demographic characteristics stratified by tertile of composite dietary antioxidant index (*N* = 29 459).

	Overall (−8.28, 117.44)	CDAI 1 (−8.28, −1.99)	CDAI 2 (−1.99, 0.89)	CDAI 3 (0.89, 117.44)	*p*
**Number of subjects**	29459	9819	9819	9821	
**Composite dietary antioxidant index**	0.31 (0.05)	−3.56 (0.02)	−0.60 (0.01)	4.26 (0.06)	**< 0.001**
**Sex**					**< 0.001**
Male	14481 (49.16)	3569 (36.35)	4670 (47.56)	6242 63.56)	
Female	14978 (50.84)	6250 (63.65)	5149 (52.44)	3579 (36.44)	
**Race**					**< 0.001**
Non‐Hispanic White	12319 (41.82)	3786 (38.56)	4199 (42.76)	4334 (44.13)	
Others	17140 (58.18)	6033 (61.44)	5620 (57.24)	5487 (55.87)	
**Age**					**< 0.001**
20–39	9741 (33.07)	2990 (30.45)	3227 (32.86)	3524 (35.88)	
40–59	9851 (33.44)	3121 (31.79)	3288 (33.49)	3442 (35.05)	
≥ 60	9867 (33.49)	3708 (37.76)	3304 (33.65)	2855 (29.07)	
**Education level**					**< 0.001**
Below high school	7015 (23.81)	3058 (31.14)	2215 (22.56)	1742 (17.74)	
High school	6761 (22.95)	2504 (25.50)	2210 (22.51)	2047 (20.84)	
Above high school	15683 (53.24)	4257 (43.36)	5394 (54.93)	6032 (61.42)	
**Marital status**					**< 0.001**
Married/living with partner	17528 (59.50)	5299 (53.97)	6043 (61.54)	6186 (62.99)	
Widowed/divorced/separated/never married	11931 (40.50)	4520 (46.03)	3776 (38.46)	3635 (37.01)	
**Smoking status**					**< 0.001**
Current Smoker	5969 (20.26)	2453 (24.98)	1805 (18.38)	1711 (17.42)	
Former Smoker	7075 (24.02)	2120 (21.59)	2419 (24.64)	2536 (25.82)	
Never	16415 (55.72)	5246 (53.43)	5595 (56.98)	5574 (56.76)	
**Alcohol consumption**					**< 0.001**
Heavy drinker	2766 (9.39)	725 (7.38)	883 (8.99)	1158 (11.79)	
Moderate drinker	5693 (19.33)	1496 (15.24)	2032 (20.69)	2165 (22.04)	
Never	21000 (71.28)	7598 (77.38)	6904 (70.32)	6498 (66.17)	
**Physical activity**					**< 0.001**
Active	17736 (60.21)	5199 (52.95)	5873 (59.81)	6664 (67.85)	
Inactive	11723 (39.79)	4620 (47.05)	3946 (40.19)	3157 (32.15)	
**Body mass index (BMI)**					**< 0.001**
Normal	8371 (28.42)	2656 (27.05)	2686 (27.36)	3029 (30.84)	
Overweight	9684 (32.87)	3117 (31.74)	3238 (32.98)	3329 (33.90)	
Obese	11404 (38.71)	4046 (41.21)	3895 (39.66)	3463 (35.26)	
**Sleep mode**					**< 0.001**
Poor	5744 (19.50)	2149 (21.89)	1842 (18.76)	1753 (17.85)	
Moderate	10767 (36.55)	3691 (37.59)	3522 (35.87)	3554 (36.19)	
Healthy	12948 (43.95)	3949 (40.52)	4455 (45.37)	4514 (45.96)	
**Diabetes**					**< 0.001**
No	25616 (86.95)	8326 (84.79)	8532 (86.89)	8758 (89.18)	
Yes	3843 (13.05)	1493 (15.21)	1287 (13.11)	1063 (10.82)	
**Hypertension**					**< 0.001**
No	18769 (63.71)	5892 (60.01)	6301 (64.17)	6576 (66.96)	
Yes	10690 (36.29)	3927 (39.99)	3518 (35.83)	3245 (33.04)	
**CVDs**					**< 0.001**
No	26377 (91.83)	8589 (90.35)	8793 (91.35)	8995 (93.46)	
Yes	3082 (8.17)	1230 (9.65)	1026 (8.65)	826 (6.54)	

*Note:* Continuous variables were expressed with means (standard error) and categorical variables were described with quantities (percentages). Continuous variables were compared in three groups using One‐Way ANOVA and categorical variables were compared using chi‐square tests. *p* < 0.05 was set as the threshold of statistical significance and marked in bold values.

Table 2 shows logistic regression analysis results assessing the association between CDAI and CVDs in the three models. In all three models, CDAI demonstrated a strong association with CVDs. Notably, in the fully adjusted model (Model 3), the OR for the CDAI 3 group was 0.80 (0.68, 0.95), in comparison to the CDAI 1 group (*p* < 0.05). Besides, the OR for the continuous variable CDAI was 0.98 (0.96, 0.99) and the result was also statistically significant. The probability of CVDs decreased as the level of CDAI increased. These findings suggest that higher CDAI scores were associated with reduced odds of having CVDs.

**TABLE 2:** Logistic regression model of composite dietary antioxidant index on CVDs.

	Model 1^a^		Model 2^b^		Model 3^c^	
	OR (95% CI)	*p*	OR (95% CI)	*P*	OR (95% CI)	*p*
**Composite dietary antioxidant index (Continuity Value)**	0.95 (0.94, 0.97)	**< 0.001**	0.95 (0.93, 0.97)	**< 0.001**	0.98 (0.96, 0.99)	**0.010**
CDAI 1	1.00	—	1.00	—	1.00	—
CDAI 2	0.89 (0.77, 1.02)	0.092	0.86 (0.74, 1.00)	0.054	1.01 (0.86, 1.19)	0.869
CDAI 3	0.66 (0.57, 0.76)	**< 0.001**	0.63 (0.54, 0.74)	**< 0.001**	0.80 (0.68, 0.95)	**0.012**

^a^ Model 1 without adjustments.

^b^ Model 2 additionally adjusted for sex, age, and race.

^c^ Model 3 additionally adjusted for education level, marital status, smoking status, alcohol level, physical activity, BMI, sleep mode, diabetes, and hypertension.

Table 3 shows the results of the stratified analysis of the association between CDAI and CVDs. The results showed a suggestive interaction effect between CDAI and alcohol consumption on CVDs (*P*‐interaction = 0.092), whereas none of the other variables had a statistically significant interaction with CDAI. Based on the results in Table 4, we found that in all three models, the lower tertile of CDAI was associated with a higher prevalence of CVDs only in the never drinker subgroup. In the final model (Model 3), the OR was 0.74 (0.60, 0.92) for the CDAI 3 groups when compared to the CDAI 1 group (*p* < 0.05).

**TABLE 3:** Stratified analysis of the associations between composite dietary antioxidant index and CVDs.

Variables	CDAI 1		CDAI 2		CDAI 3		*p*‐interaction
	OR (95% CI)	*p*	OR (95% CI)	*p*	OR (95% CI)	*p*	
**Age**							0.176
20–39	1.00	—	1.33 (0.77, 2.29)	0.304	0.73 (0.39, 1.37)	0.327	
40–59	1.00	—	0.89 (0.63, 1.25)	0.496	0.76 (0.54, 1.07)	0.116	
≥ 60	1.00	—	1.03 (0.87, 1.22)	0.732	0.83 (0.68, 1.02)	0.075	
**Sex**							0.637
Male	1.00	—	1.04 (0.84, 1.28)	0.728	0.82 (0.66, 1.01)	0.061	
Female	1.00	—	1.01 (0.82, 1.26)	0.899	0.79 (0.60, 1.05)	0.104	
**Race**							0.740
Non‐Hispanic White	1.00	—	1.05 (0.84, 1.29)	0.684	0.81 (0.65, 1.00)	0.053	
Others	1.00	—	0.92 (0.74, 1.14)	0.449	0.76 (0.58, 1.01)	0.057	
**Education level**							0.280
Below high school	1.00	—	0.94 (0.71, 1.25)	0.666	0.65 (0.49, 0.85)	0.002	
High school	1.00	—	0.98 (0.70, 1.38)	0.920	0.85 (0.61, 1.19)	0.342	
Above high school	1.00	—	1.09 (0.87, 1.36)	0.441	0.83 (0.65, 1.06)	0.139	
**Marital status**							0.284
Married/living with partner	1.00	—	0.89 (0.72, 1.11)	0.300	0.74 (0.59, 0.93)	0.010	
Widowed/divorced/separated/never married	1.00	—	1.20 (0.96, 1.49)	0.110	0.85 (0.65, 1.13)	0.260	
**Smoking status**							0.173
Current Smoker	1.00	—	0.83 (0.59, 1.17)	0.279	0.975		
Former Smoker	1.00	—	0.90 (0.70, 1.16)	0.415	0.64 (0.48, 0.85)	0.003	
Never	1.00	—	1.20 (0.94, 1.54)	0.143	0.86 (0.65, 1.14)	0.299	
**Alcohol consumption**							**0.092**
Heavy drinker	1.00	—	1.17 (0.65, 2.08)	0.601	1.13 (0.62, 2.04)	0.692	
Moderate drinker	1.00	—	1.22 (0.84, 1.77)	0.283	0.93 (0.66, 1.32)	0.689	
Never	1.00	—	0.97 (0.82, 1.15)	0.704	0.74 (0.60, 0.92)	0.007	
**Physical activity**							0.387
Active	1.00	—	1.05 (0.82, 1.33)	0.719	0.63 (0.50, 0.81)	0.263	
Inactive	1.00	—	0.98 (0.80, 1.20)	0.862	0.71 (0.56, 0.89)	0.003	
**Body mass index (BMI)**							0.140
Normal	1.00	—	1.11 (0.81, 1.51)	0.516	1.01 (0.71, 1.43)	0.956	
Overweight	1.00	—	0.80 (0.60, 1.05)	0.101	0.70 (0.52, 0.93)	0.016	
Obese	1.00	—	1.11 (0.87, 1.40)	0.399	0.76 (0.58, 0.98)	0.038	
**Sleep mode**							0.164
Poor	1.00	—	0.88 (0.65, 1.18)	0.383	0.70 (0.53, 0.93)	0.013	
Moderate	1.00	—	1.31 (1.01, 1.69)	0.045	0.94 (0.71, 1.24)	0.653	
Healthy	1.00	—	0.88 (0.68, 1.14)	0.333	0.75 (0.56, 1.00)	0.047	
**Diabetes**							0.942
No	1.00	—	1.02 (0.85, 1.22)	0.839	0.81 (0.66, 0.99)	0.037	
Yes	1.00	—	0.99 (0.75, 1.30)	0.947	0.77 (0.53, 1.13)	0.184	
**Hypertension**							0.909
No	1.00	—	0.94 (0.73, 1.21)	0.611	0.77 (0.57, 1.03)	0.074	
Yes	1.00	—	1.05 (0.86, 1.30)	0.619	0.83 (0.68, 1.01)	0.065	

*Note:* Adjusted for sex, age, race, education level, marital status, smoking status, alcohol level, physical activity, BMI, sleep mode, diabetes, and hypertension. Of note, the variables examined in this table were not adjusted. *p* < 0.10 was considered a result of the interaction as suggestive and marked in bold values.

**TABLE 4:** Stratified analysis of the associations between composite dietary antioxidant index and CVDs by alcohol consumption.

	Model 1^a^		Model 2^b^		Model 3^c^	
	OR (95% CI)	*p*	OR (95% CI)	*p*	OR (95% CI)	*p*
**Heavy drinker**						
CDAI 1	1.00	—	1.00	—	1.00	—
CDAI 2	1.15 (0.66, 2.01)	0.618	1.03 (0.56, 1.92)	0.916	1.17 (0.65, 2.08)	0.601
CDAI 3	1.08 (0.64, 1.82)	0.776	0.93 (0.54, 1.59)	0.779	1.13 (0.62, 2.04)	0.692
**Moderate drinker**						
CDAI 1	1.00	—	1.00	—	1.00	—
CDAI 2	1.12 (0.79, 1.59)	0.510	1.04 (0.72, 1.49)	0.844	1.22 (0.84, 1.77)	0.283
CDAI 3	0.88 (0.63, 1.22)	0.430	0.73 (0.51, 1.02)	0.066	0.93 (0.66, 1.32)	0.689
**Never**						
CDAI 1	1.00	—	1.00	—	1.00	—
CDAI 2	0.87 (0.75, 1.02)	0.077	0.85 (0.72, 1.01)	0.062	0.97 (0.82, 1.15)	0.704
CDAI 3	0.63 (0.53, 0.75)	**< 0.001**	0.62 (0.51, 0.75)	**< 0.001**	0.74 (0.60, 0.92)	**0.007**

^a^ Model 1 without adjustments.

^b^ Model 2 additionally adjusted for sex, age, and race.

^c^ Model 3 additionally adjusted for education level, marital status, smoking status, physical activity, BMI, sleep mode, diabetes, and hypertension.

On the basis of interaction effects, we divided participants into nine subgroups to specifically analyze the effects of CDAI and alcohol consumption on the odds of CVDs, as shown in Table 5. There was a statistically significant increased prevalence of CVDs in the CDAI 2 & never drinking subgroups and in the CDAI 1 & never drinking subgroups compared with the CDAI 3 & moderate drinking group (*p* < 0.05). The results indicate that lower CDAI scores with no alcohol consumption may increase the odds of developing CVDs and that higher CDAI levels and moderate alcohol consumption may have a potential protective effect on cardiovascular health.

**TABLE 5:** Logistic regression of CVDs for composite dietary antioxidant index and alcohol consumption subgroups.

	Model 1^a^		Model 2^b^		Model 3^c^	
	OR(95% CI)	*p*	OR(95% CI)	*p*	OR(95% CI)	*p*
Subgroup 1 (*N* = 2165)	1.00	—	1.00	—	1.00	—
Subgroup 2 (*N* = 6498)	1.27 (0.95, 1.69)	0.102	1.29 (0.95, 1.75)	0.098	1.05 (0.78, 1.40)	0.757
Subgroup 3 (*N* = 1158)	0.88 (0.54, 1.44)	0.602	0.98 (0.59, 1.66)	0.952	0.91 (0.54, 1.52)	0.706
Subgroup 4 (*N* = 2032)	1.28 (0.89, 1.85)	0.188	1.37 (0.94, 2.01)	0.103	1.26 (0.86, 1.85)	0.235
Subgroup 5 (*N* = 6904)	1.76 (1.34, 2.32)	**< 0.001**	1.80 (1.35, 2.41)	**< 0.001**	1.38 (1.04, 1.84)	**0.027**
Subgroup 6 (*N* = 883)	0.94 (0.55, 1.59)	0.808	1.03 (0.59, 1.79)	0.930	0.87 (0.51, 1.48)	0.598
Subgroup 7 (*N* = 1496)	1.14 (0.82, 1.59)	0.430	1.29 (0.90, 1.83)	0.161	0.97 (0.66, 1.41)	0.855
Subgroup 8 (*N* = 7598)	2.02 (1.56, 2.61)	**< 0.001**	2.14 (1.61, 2.86)	**< 0.001**	1.45 (1.11, 1.90)	**0.008**
Subgroup 9 (*N* = 725)	0.81 (0.53, 1.25)	0.346	0.98 (0.62, 1.57)	0.946	0.67 (0.43, 1.03)	0.069

*Note:* Subgroup 1 is the CDAI 3 and the moderate drinker; Subgroup 2 is the CDAI 3 and the never drinker; Subgroup 3 is the CDAI 3 and the heavy drinker; Subgroup 4 is the CDAI 2 and the moderate drinker; Subgroup 5 is the CDAI 2 and the never drinker; Subgroup 6 is the CDAI 2 and the heavy drinker; Subgroup 7 is the CDAI 1 and the moderate drinker; Subgroup 8 is the CDAI 1 and the never drinker; Subgroup 9 is the CDAI 1 and the heavy drinker.

^a^ Model 1 without adjustments.

^b^ Model 2 additionally adjusted for sex, age, and race.

^c^ Model 3 additionally adjusted for education level, marital status, smoking status, alcohol level, physical activity, BMI, sleep mode, diabetes, and hypertension; *p* < 0.05 was set as the threshold of statistical significance and marked in bold values.

## 4 Discussion

In this study, we investigated the association between CDAI and CVDs in US adults using data from NHANES. After adjusting for confounders, the results indicated that lower levels of CDAI were positively associated with the prevalence of CVDs. In addition, there was an interaction between CDAI combined with alcohol consumption on CVDs. The results showed that lower levels of CDAI had a greater effect on CVDs in the non‐drinking group, whereas moderate and heavy drinking appeared to reduce the deleterious effects of low levels of CDAI on CVDs. Furthermore, the combination of low CDAI levels with never drinking alcohol significantly increased the odds of CVDs. Our findings highlighted the importance of dietary interventions for the prevention of CVDs.

It is well known that dietary sources of antioxidants have a tight association with the redox system of the body [10, 35, 36]. The vitamin A inhibits oxidative stress and apoptosis in the organism [37]. A randomized controlled trial showed that vitamin C inhibits oxidative stress signaling in the body [38]. A study by Blaner et al. showed that vitamin E, in addition to being a direct antioxidant, prevents the increase of lipid peroxidation [39]. However, some of the past studies that include the above are usually based on measurements of individual pro‐oxidant or antioxidant foods and may not provide a comprehensive assessment of nutritional exposure and whole‐body redox status. The importance of the overall diet and the complex interactions between individual nutrients, rather than specific nutrients, is increasingly recognized [40, 41]. We introduced a comprehensive scoring system, the CDAI, to assess dietary antioxidant levels in this study to explore the impact of the combined effect between it and drinking status on CVDs [15]. The development of OS in the vasculature and the weakening of antioxidant defenses are the main mechanisms leading to the development of CVDs, including hypertension, atherosclerosis, aortic aneurysm, and restenosis [4, 42, 43]. OS induces changes in endothelial cells or vascular smooth muscle cells that increase the levels of NOXs expressed by both, leading to overproduction of ROS and further OS [44]. Such a state contributes to CVDs by inducing endothelial cell dysfunction and inflammation, inhibiting nitric oxide (NO) levels, promoting vascular smooth muscle cell (VSMC) proliferation, migration, and deposition of extracellular matrix (ECM) proteins, as well as altering vascular responses and vascular tone [45, 46]. In addition, excess reactive oxygen species may also activate the NF‐κB pathway and trigger systemic inflammation by increasing inflammatory mediators. Peroxiredoxin‐2 is thought of as a link between oxidative stress and inflammation [47, 48]. In contrast, dietary antioxidants may protect cardiovascular health by reducing inflammation, improving endothelial vasodilation, and increasing the production of vasodilatory compounds such as nitric oxide, among other mechanisms, thereby reducing the development of CVDs.

Previous studies have shown that alcohol consumption has been shown to have a complex and sometimes contradictory association with CVDs [20, 49]. Because of the uncertainty surrounding the effects of alcohol consumption on CVDs, the relationship between alcohol consumption and CVDs is still worth exploring. The results of our study indicated an interaction between CDAI and alcohol consumption, and further stratification showed that the association between CDAI and CVDs was significant only in the never‐drinking subgroup. Non‐drinking combined with lower CDAI scores may increase the risk of developing CVDs compared with CDAI 3 combined with moderate drinkers. This is in general agreement with the results of previous studies. A meta‐analysis of 63 intervention studies showed that moderate alcohol consumption may protection against coronary heart disease (CHD) through underlying pathophysiological mechanisms [50]. The results of another review containing 31 studies indicated that moderate alcohol consumption may reduce the risk of atherosclerosis through changes in lipid profiles and inflammation [51]. In addition, moderate alcohol consumption was associated with lower fasting insulin and HbA1c concentrations [52]. In addition, moderate alcohol consumption has been associated with improved hemostasis in the body [53]. Thus, moderate alcohol consumption may have some health benefits, and the results of a previous study have also shown that moderate alcohol consumption (1–2 cups per day for women and 2–4 cups per day for men) was negatively associated with total mortality in both men and women [54]. In addition, drinking behavior appeared to attenuate the association between CDAI and CVDs, suggesting that there may be some protective effect of alcohol consumption against the negative effects of low levels of CDAI on CVDs. Should drinking, on the other hand, be recommended to start drinking alcohol as an important risk factor for cancer worldwide? We believe that the association between drinking behavior and health should be reconsidered. The choice of alcohol consumption should be based on individual considerations and should take full account of dietary and behavioral habits and other factors that may vary with age and lifestyle, as well as not abusing alcohol and ignoring the risks of alcohol abuse.

There are numerous strengths to this study. Firstly, it is significant in that it is the first study to investigate the association between CDAI, alcohol consumption, and CVDs. Secondly, the use of CDAI allows for a comprehensive assessment of overall dietary antioxidant intake with greater accuracy. Thirdly, this study uses NHANES data analyzed under complex weighting, which more accurately reflects the health status of the U.S. population. However, certain limitations should be acknowledged. First, establishing causal relationships between CDAI, alcohol consumption, and CVDs is challenging due to the cross‐sectional character of the study. Second, individual data from NHANES may be subject to recall bias and selection bias. More large prospective studies and mechanistic studies are needed in the future to delve deeper into the associations among the three.

## 5 Conclusion

The interaction between CDAI and alcohol consumption was found in our study. High levels of CDAI combined with moderate alcohol consumption may reduce the odds of CVDs. It is recommended that individuals increase their CDAI levels by consuming antioxidant‐rich foods, thereby reducing the odds of CVDs.

## Author Contributions

Conceptualization: Yingjie Zhu, Lixin Wan; Methodology: Yingjie Zhu; Formal Analysis: Lili Zheng; Investigation: Jia Bing, Xiaoyu Teng; Writing‐Original Draft Preparation: Yingjie Zhu; Writing – review and editing: Lili Zheng, Jia Bing, Xiaoyu Teng, Pengkai Hao, Ping Song, Lixin Wan; Supervision: Lixin Wan. All authors read and agreed with the final manuscript.

## Funding

The authors received no specific funding for this work.

## Ethics Statement

Institutional Review Board approval was waived as NHANES data is deidentified and publicly available. All the participants signed the informed consent before participating in the study.

## Consent

All authors agreed with the final version of the manuscript.

## Conflicts of Interest

The authors declare no conflicts of interest.

## Data Availability

The data underlying this article are available in [National Health and Nutrition Examination Survey], at https://www.cdc.gov/nchs/nhanes/index.htm.
